# Maternal melatonin treatment rescues endocrine, inflammatory, and transcriptional deregulation in the adult rat female offspring from gestational chronodisruption

**DOI:** 10.3389/fnins.2022.1039977

**Published:** 2022-11-23

**Authors:** Natalia Mendez, Diego Halabi, Esteban Roberto Salazar-Petres, Karina Vergara, Fernando Corvalan, Hans G. Richter, Carla Bastidas, Pía Bascur, Pamela Ehrenfeld, Maria Seron-Ferre, Claudia Torres-Farfan

**Affiliations:** ^1^Laboratorio de Cronobiología del Desarrollo, Facultad de Medicina, Instituto de Anatomía, Histología y Patología, Universidad Austral de Chile, Valdivia, Chile; ^2^School of Dentistry, Facultad de Medicina, Universidad Austral de Chile, Valdivia, Chile; ^3^Centro Interdisciplinario de Estudios del Sistema Nervioso (CISNe), Universidad Austral de Chile, Valdivia, Chile; ^4^Programa de Fisiopatología, ICBM, Facultad de Medicina, Universidad de Chile, Santiago, Chile

**Keywords:** fetal programming of adult disease, melatonin, reprogramming, gestational chronodisruption, Non-Communicable Diseases (NCDs)

## Abstract

**Introduction:**

Gestational chronodisruption impact maternal circadian rhythms, inhibiting the nocturnal increase of melatonin, a critical hormone that contributes to maternal changes adaptation, entrains circadian rhythms, and prepares the fetus for birth and successful health in adulthood. In rats, we know that gestational chronodisruption by maternal chronic photoperiod shifting (CPS) impaired maternal melatonin levels and resulted in long-term metabolic and cardiovascular effects in adult male offspring. Here, we investigated the consequences of CPS on mother and adult female offspring and explored the effects of melatonin maternal supplementation. Also, we tested whether maternal melatonin administration during gestational chronodisruption rescues maternal circadian rhythms, pregnancy outcomes, and transcriptional functions in adult female offspring.

**Methods:**

Female rats raised and maintained in photoperiod 12:12 light: dark were mated and separated into three groups: (a) Control photoperiod 12:12 (LD); (b) CPS photoperiod; and (c) CPS+Mel mothers supplemented with melatonin in the drinking water throughout gestation. In the mother, we evaluated maternal circadian rhythms by telemetry and pregnancy outcomes, in the long-term, we study adult female offspring by evaluating endocrine and inflammatory markers and the mRNA expression of functional genes involved in adrenal, cardiac, and renal function.

**Results:**

In the mothers, CPS disrupted circadian rhythms of locomotor activity, body temperature, and heart rate and increased gestational length by almost 12-h and birth weight by 12%, all of which were rescued by maternal melatonin administration. In the female offspring, we found blunted day/night differences in circulating levels of melatonin and corticosterone, abnormal patterns of pro-inflammatory cytokines Interleukin-1a (IL1a), Interleukin-6 (IL6), and Interleukin-10 (IL10); and differential expression in 18 out of 24 adrenal, cardiac, and renal mRNAs evaluated.

**Conclusion:**

Maternal melatonin contributed to maintaining the maternal circadian rhythms in mothers exposed to CPS, and the re-establishing the expression of 60% of the altered mRNAs to control levels in the female offspring. Although we did not analyze the effects on kidney, adrenal, and heart physiology, our results reinforce the idea that altered maternal circadian rhythms, resulting from exposure to light at night, should be a mechanism involved in the programming of Non-Communicable Diseases.

## Introduction

In urbanized societies, regular light/dark cycles are threatened, bringing about the disarray of the internal temporal order of circadian physiological functions, called chronodisruption (Erren and Reiter, 2009; Bonmati-Carrion et al., 2014), affecting wellbeing from gestation to adulthood (Bagci et al., 2020; Hsu and Tain, 2020b; Torres-Farfan et al., 2020). Human epidemiological studies have demonstrated an association between gestational chronodisruption and adverse pregnancy outcomes (Croteau et al., 2007; Abeysena et al., 2009; Cai et al., 2019). Furthermore, maternal chronodisruption in murine models (pregnant rats exposed to chronic photoperiod shift -CPS-) induces the circadian system’s disarray in the adult male offspring, affecting metabolic and cardiovascular physiology (Varcoe et al., 2011; Ferreira et al., 2012; Galdames et al., 2014; Mendez et al., 2016, 2019; Salazar et al., 2018; Carmona et al., 2019). Regarding the mechanisms involved, the maternal and fetal circadian systems’ interaction is recognized as a significant physiological factor, in which, maternal melatonin plays a key role (Seron-Ferre et al., 2007; Mark et al., 2017; Astiz and Oster, 2018), fitting health in adulthood, as seen in chronic diseases that influence our modern society, also named Non-Communicable Diseases (NCD); with gestational chronodisruption being analogous to other human and animal models of Developmental Origin of Health and Disease -DOHaD- (Osmond and Barker, 2000; Fowden et al., 2006; Suzuki, 2018).

Melatonin is secreted primarily in the pineal gland, which is associated with several physiological systems. The main function of melatonin is to relay information to the body regarding the length of the light and dark cycles, allowing the body to respond by shifting many physiological functions (Cipolla-Neto and Do Amaral, 2018). Melatonin interacts with intracellular molecules and presents receptor-mediated actions that result from the interaction with both membrane and nuclear receptors. These receptors are widely expressed in the central nervous system and peripheral organs since embryo development, which explains the wide actions of this hormone (Cipolla-Neto and Do Amaral, 2018; McCarthy et al., 2019).

During gestation, numerous maternal physiological functions are circadian and provide potentially redundant signals for fetal circadian rhythm entrainment, where maternal melatonin is one of the few hormones crossing the placenta unaltered; therefore, melatonin may play a key synchronizing role (Seron-Ferre et al., 2007; Reiter et al., 2014; Torres-Farfan et al., 2020; Astiz and Oster, 2021). Interestingly, melatonin levels are modified during pregnancy, contributing to maternal changes adaptation (Reiter et al., 2014; McCarthy et al., 2019; Cipolla-Neto et al., 2022), since in human pregnancy, maternal melatonin levels increase progressively from 24 weeks of gestation until term (Tamura et al., 2008a); in rats, maternal melatonin’s peak increases about threefold from day 7 of pregnancy to term and then drops to non-pregnant levels (Tamura et al., 2008b; Bates and Herzog, 2020). Also, melatonin probably prepares the fetus for birth, since the fetuses in almost all the species studied did not produce melatonin (Nakamura et al., 2001; Tamura et al., 2008b), however, all the fetal tissues studied from several species expressed melatonin receptors (Torres-Farfan et al., 2020).

Maternal models of pinealectomy, shifted photoperiod or constant light have in common the perturbation of melatonin rhythm during gestation and showed long-term consequences in the offspring. Interestingly, when the mother received a daily dose of melatonin during the subjective night, some effects were reversed, supporting a causal role of melatonin deprivation on the phenotypes observed (Ferreira et al., 2012; Vilches et al., 2014; Tain et al., 2017b). Emerging evidence supports that the effects of melatonin manipulation are closely related to other proposed mechanisms contributing to the developmental programming of various chronic NCDs’, including pro-inflammatory status (Hsu and Tain, 2020a); many of these processes could entail a low-grade inflammation state, as suggested by the up-regulation of TNF-a, IL-1b, and IL-6 in various tissues from rat and other animal models of chronodisruption by sleep deprivation (Carroll et al., 2015; Wright et al., 2015).

Low-grade inflammation has been related to the development of chronic diseases (Lopez-Lopez et al., 2015). Although there is scarce evidence linking gestational chronodisruption with low-grade inflammation, our previous finding shows that gestational chronodisruption promotes an increase of pro-inflammatory cytokines in male offspring (Mendez et al., 2019). Therefore, melatonin alteration could be a critical factor for fetal programming chronic diseases triggered by gestational chronodisruption. In this context, we hypothesized that irregular circadian melatonin rhythm during gestation could be a determinant factor for fetal programming in the adult female offspring; melatonin administration to mothers would reverse or prevent the changes induced by CPS in the adult offspring.

## Materials and methods

All animal care and experimental procedures were approved by the Committee of bioethics of the Universidad Austral de Chile and carried out following international standards of the Guide for the Care and Use of Laboratory Animals of the Institute for Laboratory Animal Research of the National Research Council.

### Animals and experimental procedures

**FIGURE 1:**
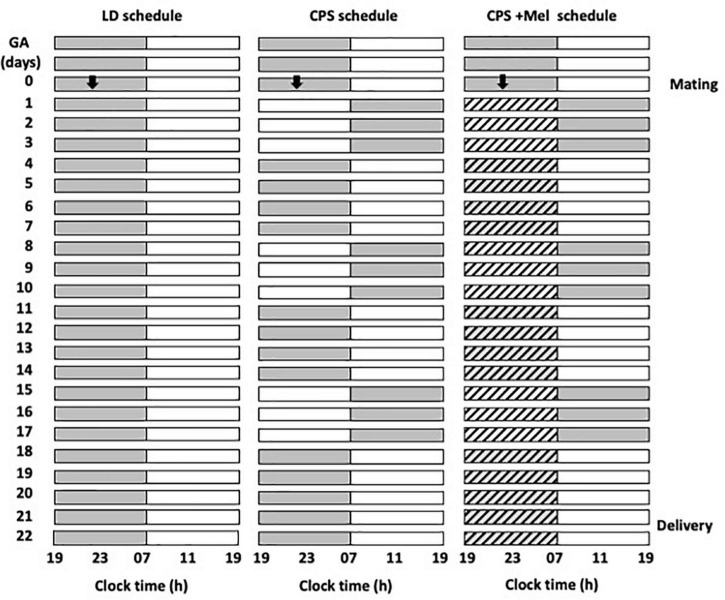
Schematic representation of the photoperiod protocols used throughout gestation. Three cohorts of female rats raised and maintained in photoperiod 12-h light and 12-h dark were mated and separated into three groups: LD photoperiod, CPS photoperiod, and CPS+Mel, corresponding to females under CPS that received melatonin in drinking water during subjective night (19:00–07:00). In CPS, the photoperiod was shifted by 12 h for three consecutive cycles, after which the dark phase was extended for 12 h to restore the original photoperiod for a further 4 days. The melatonin treatment was available from GA 1 until delivery (GA, gestational age).

We obtained Sprague–Dawley rats from Charles River (CRL International Inc., Kingston, NY) and housed them in our facility under standard photoperiod (12:12 h light-dark cycle), light on at 07:00 A.M.; ∼400 lx at the head level; controlled temperature (20 ± 2°C), food and water *ad libitum*. Then, young females (90–110 days of age) were mated during the night, and the next morning at 09:00 h, vaginal smears were made to determine the presence of sperm and to delimit zero days of gestation or embryonic day (E0), stage at which pregnant females were randomly allocated into three groups and maintained under the following photoperiod protocols (Figure 1):

LD: Pregnant rats maintained during gestation in a standard photoperiod 12:12 h (light: Dark cycle; lights on at 07:00 A.M.).

Chronic photoperiod shifting: Pregnant rats were exposed to chronic photoperiod shifting (simulating rotating shift work) until 18 days of gestation, then, pregnant dams returned to normal LD photoperiod until delivery (Mendez et al., 2016). Briefly, pregnant females were exposed to lighting schedule manipulation every 3–4 days reversing completely the photoperiod. The photoperiod reversal occurred at the night of day 0 of gestation, so that lights rather than going off at 1,900 h, remained on until 0700 h of day 2. At 18 days of gestation, the mothers returned to standard photoperiod (12:12, lights on at 0700 h) and continued in this photoperiod thereafter (Figure 1).

CPS + Mel: Pregnant rats exposed to chronic photoperiod shifting were treated with melatonin in the drinking water between 19:00 and 07:00. Briefly, a bottle of water with melatonin (CPS + Mel) or without melatonin (CPS) was replaced from 0 days of gestation until 18 days of gestation. Melatonin stock solution was prepared by dissolving 1 g of melatonin in 100 mL ethanol plus 400 mL of water. The solution in bottles was prepared to dilute 1 mL of stock solution in 1,000 mL of water, therefore the final concentration of melatonin was 2 μg/mL to achieve a dose of 150–165 μg/kg of weight. The work solution was prepared fresh every 2 days and was put in a flask protected from light. In the three groups of pregnant females, we measured water consumption between 1,900 and 0700 h. The bottles were weighed before and after the change at 1,900 and 0700 h, respectively. As reported previously we did not find differences in water consumption between the groups (LD: 29.69 ± 0.94; CPS: 30.21 ± 1.07; CPS ± Mel: 30.95 ± 0.59 ml), similar to those reported previously for us in other models in which we use melatonin treatment during gestation (Vilches et al., 2014).

Here, we explored the effects of CPS and CPS + Mel on maternal rhythms through telemetric studies, and then we analyzed the impact on adult female offspring tissues’ gene expression.

### Telemetric studies in pregnant dams

In adult female rats kept under control 12-h light 12-h dark photoperiod, we implanted transponders in the abdominal cavity (ER-4,000 energizer; Mini mitter Co., Inc., Bend, OR, USA) following the implantation procedures suggested by the manufacturers as we reported previously (Mendez et al., 2016). A total of 48 h before the surgery, the animals received a carprofen gel treatment that delivered an effective dose of 5 mg/kg/day (MediGel CPF; ClearH2O, Westbrook, ME, USA). On days 7–10 after surgery, the implanted females were mated during the night, and the following day at 09:00 h, vaginal smears were made to determine the presence of sperm. Females were randomly allocated into two groups CPS and CPS + Mel. Of Note, the cohort of LD control mothers used here to compare telemetric study was previously published (Mendez et al., 2016). For each experimental group of mothers, CPS (*n* = 7) and CPS + Mel (*n* = 5), we recorded the pregnant female individually in conditioned rooms placing home cages on top of the ER-4,000 energizer/receivers. Data were collected at 15-s intervals during the pregnancy until 24 h past delivery. The CPS and CPS + Mel mothers returned to standard photoperiod at 18 days of gestation; hence the delivery happened in LD conditions. As we observed previously, the transponder did not affect the gestation progression and the litter’s size.

### Adult female offspring evaluation

The second cohort of pregnant females, used in the current study, was obtained as described above LD (*n* = 6), CPS (*n* = 7), and CPS +Mel (*n* = 6) mothers, and their female offspring were used in the experiments described below to evaluate hormones and gene expression.

### Blood collection and hormones analysis

At 90 days of age, the female’s offspring from each pregnancy condition were euthanized at 11:00 and 23:00 (*n* = 6–7 per clock time, one sister in the AM period and the other PM) for tissue collection. The animals were anesthetized with isoflurane (2.0–3.0%). A midline incision and blood sample were collected from the vena cava and administered an overdose of sodium thiopental (150 mg/kg). Serum from blood samples was stored at −20°C to measure corticosterone, melatonin, and cytokines (IL1a, IL6, and IL10). Finally, the heart, kidney, and adrenal glands were collected and stored in RNAlater (Ambion Inc., Austin, TX, USA) for gene expression study.

### Gene expression analyses in adult female offspring

At 90 days of age, the female’s offspring were euthanized at 11:00 and 23:00 (*n* = 6–7 per clock time) for blood and tissue collection. The animals were anesthetized with isoflurane (2.0–3.0%). A midline incision and blood sample were collected from the vena cava and administered an overdose of sodium thiopental (150 mg/kg). Serum from blood samples was stored at −20°C to measure corticosterone, melatonin, and cytokines (IL1a, IL6, and IL10). Finally, the heart, kidney, and adrenal glands were collected and stored in RNAlater (Ambion Inc., Austin, TX, USA) for gene expression study.

### RNA extraction and relative gene expression was determined using quantitative real-time PCR analysis

Relative gene expression was determined using quantitative real-time PCR (RT-qPCR). According to the manufacturer’s instructions, total RNA was extracted from female offspring (heart, kidney, and adrenal gland) using the SV total RNA isolation system (Promega, Fitchburg, WI, USA). The SV Total RNA isolation system includes a digestion step with deoxyribonuclease to rule out contamination with genomic DNA. About 2.0 μg of total RNA was reversed transcribed using random primers (50 ng; Promega), 10 mM dNTPs (Promega), 0,1 M DTT (Promega), and 200 IU M-MLV reverse transcriptase (Invitrogen Corp., Carlsbad, CA). RT-qPCR was performed using sense and antisense primers (Table 1), H2O PCR grade, cDNA, and KAPA SYBR FAST qPCR Master Mix (Kapa Biosystems, Inc., Wilmington, MA, USA). The quantitative PCR was carried out in a Rotor-Gene Q real-time platform. After the final cycle, a melting curve analysis was performed on each sample to ensure that a single product was obtained. Relative amounts of all mRNAs were calculated by the comparative cycle threshold method ΔΔCt using the equation 2-ΔΔCt. A total of 18S-rRNA (adrenal gland) and GADPH (heart and kidney) were used as the housekeeping gene.

**TABLE 1:** Primers used for RT-qPCR studies.

Symbol	Gene bank	Primer sequence (5′->3′) Fw/Rv	Amplicon length (bp)	Annealing temperature (°C)
*Bmal*	NM024362.2	CCGATGACGAACTGAAACACCT / T GCAGTGTCCGAGGAAGATAGC	215	64
*Per-2*	NM 031678	CACCCTGAAAAGAAAGTGCGA / CAACGCCAAGGAGCTCAAGT	148	62
*Star*	NM_031558	AGAAGGAAAGCCAGCAGGAGA/ TCTCCCATGGCCTCCATG	147	60
*Hsd11b1*	NM_017080.2	CCCTGTTGGACGGCAGTTAT/ TCTTCCGATCCCTTTGCTGG	150	60
*Hsd11b2*	NM_017081.2	TCTTTGGTGCACTTGAGCTG/ CTGGATGATGCTGACCTTGA	206	60
*Dax1*	NM_053317.1	TCCAGGCCATCAAGAGTTTC/ TGTGCTCAGTGAGGATCTGC	171	60
*SF1*	NM_001191099.1	CGCCAGGAGTTTGTCTGTCT/ ACCTCCACCAGGCACAATAG	185	60
*Nur77*	NM_024388.2	GCGGAACCGCTGCCAGTTCT/ GCATCTGGGGGCTGCTTGGG	132	60
*Mt1*	NM_053676.2	CTCACTCTGGTGGCCTTGG/ AACTGCGCAGGTCACTGG	206	60
*Mt2*	NM_001100641.1	CTCACTCTGGTGGCCTTGG/ AACTGCGCAGGTCACTGG	250	60
*Egr1*	NM_012551.2	CACGTCTTGGTGCCTTTG/ CTCAGCCCTCTTCCTCATC	143	64
*PPara*	NM_013196	ACTATGGAGTCCACGCATGTG/ TGTCGTACGCCAGCTTTAGC	75	64
*Pgc1a*	NM_031347.1	AGAAGCGGGAGTCTGAAAGG/ CAGTTCTGTCCGCGTTGTG	116	64
*Kcnip2*	NM_020094	GAACAGACCAAGTTCACACG / TGAAGAGAAAAGTAGCATAGTTG	157	60
*Adra 1 a1*	NM_017191.2	CGAATCCAGTGTCTTCGCAG / ACCATGTCTCTGTGCTGTCCC	100	60
*Hmga1*	NM_139327.1	TGAAGTGCCAACCCCGAA/ CTCCTCTTCCTCCTTCTCC	142	60
*Nr3c1*	NM_012576.2	ACAGCTCACCCCTACCTTGGT / CTTGACGCCCACCTAACATGT	134	60
*Nr3c2*	NM_013131.1	CCAAAGGCTACCACAGTCTC/ TCCCAGACCGACTATTGTCT	240	60
*Kall*	NM_031523.1	GCATCACACCTGACGGATTG / GGCCTCCTGAGTCACCCTTG	171	60
*Renin*	NM_012642.4	GCTACATGGAGAATGGGACTGAA / ACCACATCTTGGCTGAGGAAAC	79	60
*Cox2*	NM_017232.3	TGTATGCTACCATCTGGCTTCGG / GTTTGGAACAGTCGCTCGTCATC	94	60
*At1r*	NM_031009.2	CAAAAGGAGATGGGAGGTCA / TGACAAGCAGTTTGGCTTTG	254	60
*Atp1a1*	NM_012504.1	CCCAAAACGGACAAACT / GCACTACCACGATACTGAC	274	60
*Ncc*	NM_019345.3	GAGAACGGCACACCCATTG / GACAAGAAAGAACAGCACCTGG	135	60
*Enac*	NM_031548.2	GCTGTTCTCCCAAGTGTCGGAA / CATCTCGAAGATCCAATCCTGG	107	60
*Hprt*	NM_012583.2	CCATCACATTGTGGCCCTCT / TATGTCCCCCGTTGACTGGT	166	60
*Gapdh*	NM_017008.4	CTCCCTCAAGATTGTCAGCA/ CCACAGTCTTCTGAGTGGCA	140	60

### Corticosterone, melatonin, and cytokines measurements in adult female offspring

The daily differences in corticosterone, melatonin, and cytokine were measured in plasma. We use two Milliplex MAP, Rat Stress Hormone and Rat Cytokine/Chemokine panels (cat. No. RSHMAG-69K and RECYTMAG-65K, respectively. Merck Millipore, Billerica, MA, USA). Samples were centrifuged at 13,000 *g* for 5 min before analysis. The assay was performed according to the manufacturer’s instructions. Plates were read using a MAGPIX plate reader and analyzed using x PONENT Software (Merck Millipore, Billerica, MA, USA). The inter-assay coefficients for corticosterone and MELATONIN were 3.9 and 7.4%, respectively. Values were expressed as ng/ml for corticosterone and pg/ml for cytokines and melatonin. Rat Cytokine/Chemokine panels were used to measure IL1a, IL6, and IL10; the current kit also allowed us to measure IL1b; TNFA, EGF, and interferon-gamma; however, in our hands, the CV was higher for these analytes and did not allow us to determine differences between the groups, at least in the clock times analyzed.

### Statistical analyses

Data are expressed as mean ± SEM. Telemetric recordings containing 15-min data collections were analyzed by cosinor using the *El Temps* program (developed by Toni Diez, Universidad de Barcelona, Barcelona, Spain). This method tests whether the data fit the cosine function with a period of 24 h as follows: Vt MA cos 15 (t-?), with Vt is the value of the variable (temperature, activity, or heart rate) at time *t*; the acrophase (an hour at which the variable reaches the maximum value), M the mesor (average of the variable over 24 h), and A the amplitude (the difference between the value of the variable at and the mesor). Also, the program performs actograms for each animal and each variable. Parameters (mesor amplitude and acrophase) of the cosinor equations fitting 24-h rhythms (*P* < 0.05) in individuals were compared by an ANOVA and a Tukey’s test. AM–PM daily changes were analyzed using an unpaired *t*-test. Treatment effects were analyzed by two-way ANOVA using Tukey’s multiple comparisons test as a *post-hoc* test. Statistical analyses were performed using GraphPad Prism version 8.00 for Mac (GraphPad Software Inc., San Diego, CA, USA).^1^ Results were considered significant at *P* < 0.05.

## Results

### Effects of gestational chronic photoperiod shifting and CPS + mel treatment on maternal circadian rhythms and pregnancy outcomes

As reported previously, pregnant rats under LD photoperiod displayed circadian oscillations of locomotor activity, temperature, and heart rate, with acrophases positioned in the dark period (Mendez et al., 2016). Here, we confirm that chronic phase shift during gestation abolished circadian rhythms of locomotor activity, temperature, and heart rate, results that were like those reported previously by us. Instead, prenatal melatonin administration during the subjective night maintains circadian rhythms for almost 11 days in CPS + Mel mothers, with slight differences in the acrophases between the first and second weeks of gestation (Table 2). Besides, we observed that CPS protocol prolonged the gestation by almost 12 h, thus shifting birth clock hours, translating into an increase in newborn weight. However, differences in litter size and sex distributions were not observed (Table 3).

**TABLE 2:** Effect of CPS + Mel on the acrophase (clock time hours) of maternal circadian rhythms measured telemetrically (Mean ± SEM).

	LD (*n* = 5)^&^			CPS + Mel (*n* = 5)		
	GW 1	GW 2	GW3	GW 1	GW 2	GW 3
Activity	00.58 ± 0.37	01.25 ± 0.44	03.85 ± 0.48*	00.56 ± 0.24	02.94 ± 0.11*^#^	–
Temperature	23.60 ± 0.27	00.42 ± 0.51	02.75 ± 0.36*	01.80 ± 0.22**^#^**	04.22 ± 0.8*^#^	–
Heart rate	23.44 ± 0.28	00.77 ± 0.41	03.11 ± 0.42*	24.17 ± 0.17**^#^**	03.68 ± 0.21*^#^	–

LD, mothers under light-dark cycle 12/12; GW, gestational week; SEM, standard error of the mean. *Different from the first week of gestation (*P* < 0.05, two ways ANOVA, and Tukey).

^#^Different from LD. ^&^Data from LD mothers has already been published (Mendez et al., 2016).

**TABLE 3:** Effect of gestational chronodisruption and melatonin supplementation on gestation length, maternal weight gain, and newborn variables.

	LD (*n* = 5)^&^	CPS (*n* = 7)^#^	CPS + Mel (*n* = 5)
Gestation length (days)	21.64 ± 0.03	22.12 ± 0.10*	22.03 ± 0.02
Maternal weight gain (g)	131.0 ± 10.2	126.0 ± 6.2	155.50 ± 7.90
Liter size (number)	13.20 ± 1.0	13.80 ± 0.97	15.5 ± 0.80
Newborn body weight (g)	6.50 ± 0.10	7.38 ± 0.10*	7.28 ± 0.22
Sex distribution (M/F)	28/32	45/51	30/32

LD, mothers under light-dark cycle 12/12; CPS, mothers under chronic photoperiod shift throughout gestation; GW, gestational week; SEM, standard error of the mean. *Different from LD (*P* < 0.001, unpaired t-test). ^&^Data from LD mothers has already been published (Mendez et al., 2016).

^#^Here we include data from CPS (*n* = 4) that has already been published (Mendez et al., 2016) plus three animals to complete *n* = 7.

### Daily differences in hormones and cytokines in adult female offspring

**FIGURE 2:**
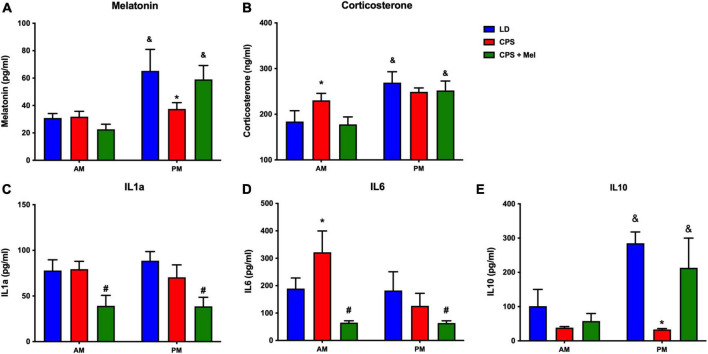
Effect of maternal melatonin supplementation on AM–PM levels of plasma melatonin, and corticosterone, in female 90-day-old offspring **(A)** and plasma cytokine levels **(B)**. Plasma was collected at 11 and 23 h (*n* = 6–7 per clock time) in females gestated in LD (blue bars), CPS (red bars), and CPS + Mel (green bars) conditions. Data are mean ± SEM. *Different to LD and CPS+Mel, ^#^Different LD to CPS (*P* < 0.05 two-way ANOVA and Tukey); ^&^Different to AM period (*P* < 0.05 unpaired *t*-test; two-way ANOVA, and Tukey).

Adult female offspring from LD, CPS, and CPS + Mel gestations were studied at 11:00 h and 23:00 h. We observed that LD females display a daily difference in melatonin and corticosterone levels. Instead, we did not find AM–PM differences in CPS female offspring at 90 days old; these levels were restored in female offspring CPS + Mel protocol (Figure 2A). Next, we evaluated a set of cytokines in plasma. CPS exposition changes the plasma levels of cytokines in the female offspring. We observed an increase in pro-inflammatory cytokines between the cytokines we could evaluate. We did not find AM–PM differences like than those reported previously for CPS male offspring; these levels were restored in female offspring CPS + Mel. In addition, treatment of maternal melatonin during pregnancy modified the cytokines significantly in the offspring, decreasing IL1a and IL6 and restoring the levels of IL10 comparable to those observed in adult female offspring gestated in LD conditions (Figure 2B).

### Effects of prenatal melatonin administration on gene expression in adult female offspring

Females’ offspring from different experimental conditions (LD, CS, and CPS + Mel) were raised in standard photoperiod. At 90 days of age, the body weight was not affected by gestational chronodisruption, although the female offspring from the mother treated with melatonin (CPS + Mel) displayed a slightly decreased body weight (LD 300.0 ± 24.2 g; CPS 310.1 ± 22.5 g; CPS + Mel: 283.8 ± 23.2. CPS + Mel; *p* < 0.05; two way-ANOVA, and Dunn’s multiple comparisons test).

**FIGURE 3:**
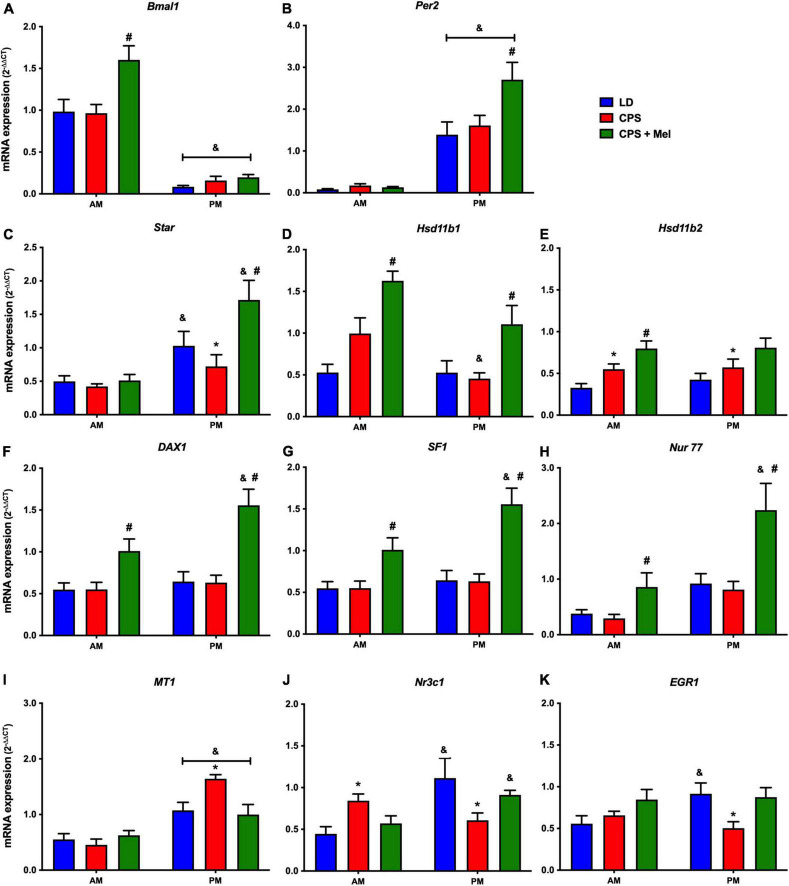
Gene expression in the adrenal gland from adult female offspring. Adrenal glands were collected at 11 and 23 h (*n* = 6–7 per clock time) in females gestated in LD (blue bars), CPS (red bars), and CPS + Mel (green bars) conditions. Data are mean ± SEM. *Different to LD and CPS+Mel, ^#^Different LD to CPS (two-way ANOVA and Tukey). ^&^Different to AM period (unpaired *t*-test; two-way ANOVA; and Tukey). Significant differences were ser when *p* < 0.05.

**FIGURE 4:**
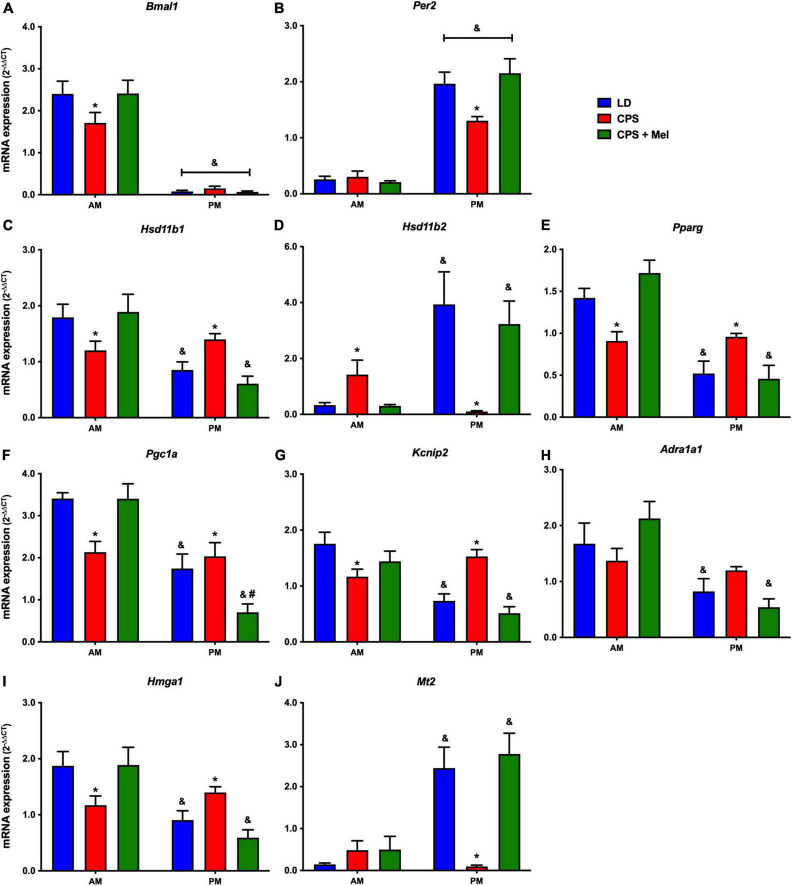
Gene expression in the heart from adult female offspring. The left ventricle was collected at 11 and 23 h (*n* = 6–7 per clock time) in females gestated in LD (blue bars), CPS (red bars), and CPS + Mel (green bars) conditions. Data are mean ± SEM. *Different to LD and CPS + Mel, ^#^Different LD to CPS (two-way ANOVA and Tukey). ^&^Different to AM period (unpaired *t*-test; two-way ANOVA, and Tukey). Significant differences were ser when *p* < 0.05.

**FIGURE 5:**
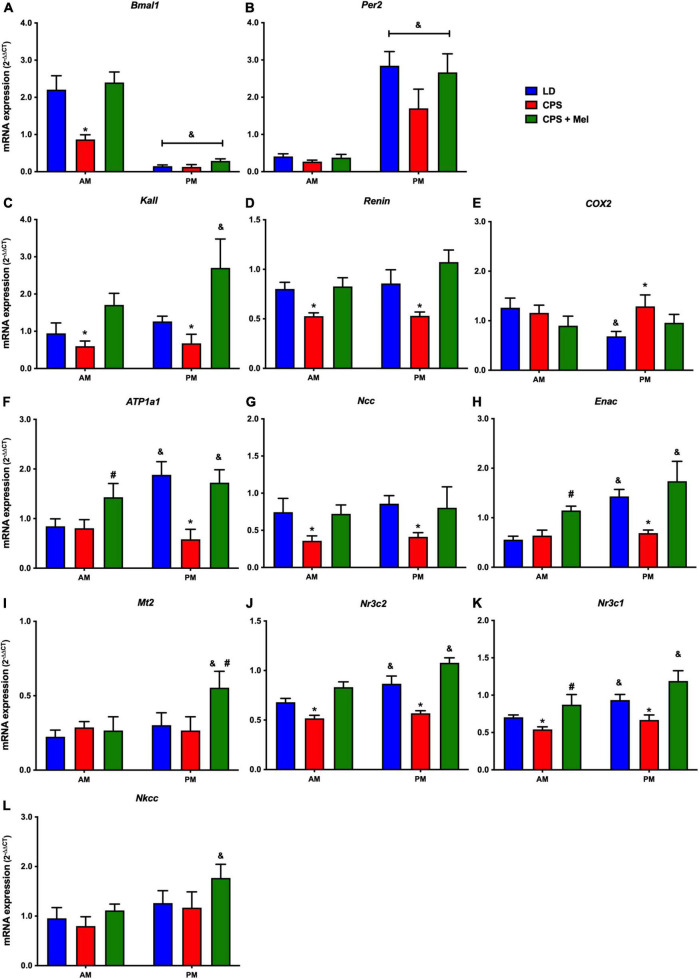
Gene expression in the kidney from adult female offspring. The whole kidney was collected at 11 and 23 h (*n* = 6–7 per clock time) in females gestated in LD (blue bars), CPS (red bars), and CPS + Mel (green bars) conditions. Data are mean ± SEM. *Different to LD and CPS + Mel, ^#^Different LD to CPS (two-way ANOVA and Tukey). ^&^Different to AM period (unpaired *t*-test; two-way ANOVA, and Tukey). Significant differences were ser when *p* < 0.05.

Next, we studied a group of selected functional genes, including clock genes, receptors, and enzymes, in three relevant peripheral clocks (heart, kidney, and adrenal glands). Females gestated under CPS conditions maintained daily differences in Per2 and Bmal1, and slight differences in expression levels were found in CPS + Mel offspring (Figures 3A,B, 4A,B, 5A,B). These results are consistent with our previous findings in male offspring at the same age (Mendez et al., 2016, 2019; Salazar et al., 2018). Regarding the genes evaluated, some clock-controlled or interacting genes involved in adrenal, heart, and kidney function were altered by CPS in female offspring.

In the adrenal gland, differences in mRNA expression between the LD and CPS were detected for Star (corticosterone synthesis), Hsd11b2 (corticosterone conversion), Nr3c1 (glucocorticoid receptor), Mt1 (melatonin receptor), and Egr1 (transcriptional regulator) genes. Maternal melatonin treatment during CPS gestation increased the relative expression of core clock genes (Bmal1 and Per2), genes involved in corticosterone production (Star), conversion (Hsd11b1, Hsd11b1-2), and transcriptional regulation genes (Dax1, Nur77, Nr3c1, and Sf1) (Figures 3A–K).

In the heart, several genes were affected in the offspring exposed to CPS, including Bmal1, Per2, and Pparγ (clock-controlled gene), Mt1, Adra1a1 receptors, and genes implicated in cardiac-endocrine regulation, such as Hsd11b1, and Hsd11b2 enzymes, Hmga1 (a non-histone chromatin protein) and Kcnip2 (a voltage-gated potassium channel-interacting protein). Interestingly, melatonin treatment during pregnancy restored normal daily expression in all genes mentioned (Figures 4A–J).

In the kidney, genes such as Atp1a (Na+/K+ ATPase alfa subunit), ENaC (reabsorption of sodium ions), Renin (key RASS component), Kallikrein (blood pressure regulator), Nr3cl (glucocorticoid receptor), and Nr3c2 (mineralocorticoid receptor) were affected by gestational chronodisruption in the CPS; melatonin treatment during gestation re-establishes the expression levels in all cases (Figures 5A–L).

These results strongly support the role of maternal melatonin as a critical photoperiodic signal to the fetus, intrinsically involved in organ development and programming since maternal melatonin treatment affected the relative expression of at least 75% of total genes evaluated and reverted 60% of them to control levels.

## Discussion

During gestation, maternal melatonin levels play an essential role in the factors that influence fetal development and maternal physiology (Reiter et al., 2014; Tain et al., 2017b; McCarthy et al., 2019). Disturbance of maternal melatonin in different models of gestational chronodisruption triggers adverse effects on the mother and the offspring (Ferreira et al., 2012; Salazar et al., 2018; Nehme et al., 2019). Previously, we observed that in a rat model, circadian rhythm perturbation in the mother induces deleterious effects in the male offspring (Mendez et al., 2016, 2019; Carmona et al., 2019; Halabi et al., 2021). In this study, we examined whether prenatal melatonin treatment could prevent short and long-term gestational effects of gestational chronodisruption on maternal rhythms and rescue the possible effects in female offspring at 90 days old.

The gestational chronodisruption, which involves melatonin decrease/absence, has been reported as a predictor of increased chronic disease risk. Indeed, pregnancy is the most challenging developmental stage encountered by a mammal throughout its life, and, therefore, healthy pregnancy seems crucial to preventing NCD in the offspring (McCarthy et al., 2019; Nehme et al., 2019; Hsu and Tain, 2020a; Gomes et al., 2021). In pregnant rats, we previously reported that CPS, modified daily rhythms of corticosterone, aldosterone, melatonin, and blunted circadian rhythms of locomotor activity, temperature, and heart rate (Varcoe et al., 2011; Mendez et al., 2016; Gatford et al., 2019). Here, we confirm that gestational CPS modified the maternal circadian rhythms, increasing gestational length by almost 12-h; effects that were prevented by maternal melatonin administration.

Even though an increase in abortions and premature birth have been reported in shift worker women (Abeysena et al., 2009; Nehme et al., 2019), we did not observe changes in the litter size, which is in line with other animal models of circadian disruption during gestation (Varcoe et al., 2011; Smarr et al., 2017; Gatford et al., 2019). In addition, although the effects of circadian disruption on litters vary between different models, a recent study demonstrated that the chronodisruption during gestation alters placental signaling in mice without changes in embryo count, placental weight, or fetal sex ratio, nevertheless enhancing the placental immune cell expression, programming a pro-inflammatory tissue environment (Clarkson-Townsend et al., 2021).

Under CPS conditions, we found a slight difference in gestational length translating into newborn weight changes. Melatonin has been described as a factor of parturition time in some species (McCarthy et al., 2019; Amano et al., 2020); however, we did not observe a significant difference in gestation length and birth time between the CPS and CPS + Mel groups. The physiological increase in melatonin synthesis throughout pregnancy can be related to health outcomes since maternal melatonin levels are reduced in the presence of gestational complications such as intrauterine growth restriction, preeclampsia, and eclampsia (Cipolla-Neto et al., 2022); however, the mechanism by which maternal serum melatonin levels change according to gestational weeks and maternal rhythms along gestation remains to be determined. In our study, the circadian rhythms of locomotor activity, temperature, and heart rate were sustained until the second week of gestation in animals with maternal melatonin treatment. Maternal melatonin supplementation was insufficient to maintain the maternal circadian rhythms until the end of gestation. Although maternal treatment supplies the melatonin absent during the CPS, our treatment could not resemble the physiological increase level observed in normal gestation.

Low melatonin levels during pregnancy are not only a complication to the mother but also influence fetal programming regarding fetus development, energy metabolism, cognition, and behavior until later in life (Ferreira et al., 2012; Motta-Teixeira et al., 2018; Hsu and Tain, 2020b). Moreover, as already described by us and others, melatonin suppression by exposure to constant light (LL) or maternal pinealectomy has overall outcomes in the offspring’s health. For instance, melatonin suppression disturbs the circadian rhythm of clock gene expression in the non-human primate fetal SCN (Seron-Ferre et al., 2007). At the same time, it modified gene expression in rat fetuses’ adrenal, heart, and hippocampus clocks (Mendez et al., 2012; Galdames et al., 2014; Vilches et al., 2014) and impaired the synchronization of temperature with the external LD cycle in newborn sheep (Torres-Farfan et al., 2008). Here, we evaluated the long-term effects of gestations under CPS and CPS + Mel on different outcomes in the adult female offspring. Gestational chronodisruption modified day/night differences of melatonin and corticosterone in adult females at 90 days old, which were restored by prenatal melatonin treatment. Corticosterone and melatonin are directly implicated in the temporal organization of daily rhythms, transducing rest/activity, and light/dark rhythms (da Silveira Cruz-Machado et al., 2017).

Consequently, chronic phase shift during gestation generated chronodisruption in adult offspring females, which could be translated into an increased risk of disease metabolic and cardiovascular, even in reproductive effects, since the link between the reproductive system and melatonin in different stages of life (Cipolla-Neto et al., 2022).

Regarding melatonin levels in the offspring, we are unknown whether CPS programmed the pineal gland directly or whether the absence of night/day difference in corticosterone might impact the melatonin levels; even though the physiological interaction between the adrenal and pineal gland is poorly understood, the evidence demonstrated that the rat pineal gland is capable of perceiving the entrance of corticosterone peak before darkness to regulate the nocturnal synthesis of melatonin, through a cooperative transcriptional program; hence corticosterone rhythm is an essential regulator of pineal function that could modulate the amplitude of melatonin peak in nocturnal animals (da Silveira Cruz-Machado et al., 2017). On the other hand, a more rapid utilization of melatonin could be another possible consequence that requires further investigation. The adrenal gland is an essential peripheral circadian oscillator synchronizing other peripheral organs during fetal and adult life. Studies in non-human primates and rat fetuses demonstrated that maternal melatonin is critical in fetal growth and adrenal gland development and entrain fetal circadian rhythms *in vivo* and *in vitro* (Torres-Farfan et al., 2006, 2011; Mendez et al., 2012). Recent evidence confirms that synchronization of adrenal clock genes and corticosterone rhythm appears early during development and depends on maternal environmental cues until the postnatal period (Roa et al., 2017). Previously we observed that the suppression of the maternal melatonin showed effects such as intrauterine growth retardation (IUGR), differential expression of several mRNAs, and altered corticosterone rhythm in the fetal adrenal gland; with many of these changes being reversed when the mother received a daily dose of melatonin during the subjective night (Torres-Farfan et al., 2011).

Programming of the fetal adrenal gland is related to permanent modifications of stress responsiveness in the offspring from gestational CPS (Salazar et al., 2018); moreover, it might influence the onset of metabolic and cardiovascular diseases. Here, we observed that maternal CPS exposure resulted in changes in gene expression in the adrenal gland in female offspring, as reported previously in male offspring; such changes in mRNA expression affect genes involved in adrenal steroids production, transcription factors, and melatonin responsive genes. Consistent with the maternal melatonin’s role in the offspring’s adrenal gland, maternal melatonin treatment reversed the effects on adrenal gland mRNA expression and restored AM–PM differences plasma corticosterone levels in female CPS + Mel, absent in CPS offspring. Although the specific mechanism by which melatonin changes gene expression is unclear, the evidence available has demonstrated the role of melatonin in this organ, which includes effects on clock genes expression and steroidogenic enzymes (Valenzuela et al., 2008; Torres-Farfan et al., 2011; Seron-Ferre et al., 2017).

As reported previously, another fetal organ impacted by maternal chronodisruption is the heart (Galdames et al., 2014). In this context, it is worth mentioning that gestational chronodisruption by constant light has been reported to modify cardiac gene networks in fetal hearts, including several established cardiovascular disease markers and persistent transcriptional changes in male offspring with left ventricular hypertrophy in adult life (Galdames et al., 2014). Here, genes such as Adra1a and Kcnip2, involved in proliferation, contractility, and electrophysiological activity, were also modified in adult females gestated under CPS. We do not know the effects of CPS on cardiac physiology because the cardiac function was not evaluated in adult females; however, we previously reported that gestational CPS impacts the heart rate variability and blood pressure in male offspring, in which significant changes in mRNA expression of genes involved, as well, in proliferation, contractility, and electrophysiological activity were observed in fetus and adult offspring (Mendez et al., 2016, 2019). Interestingly, like that observed in the gene expression in the adrenal gland, prenatal melatonin treatment modulated and restored the AM/PM differences in CPS + Mel females in genes involved in metabolism and cardiac steroidogenesis.

The circadian system contributes to time of day differences in cardiovascular parameters; therefore, the timing of these endogenous rhythms can be altered by changes in photoperiod (Vandewalle et al., 2007). We recently reported fetal programming of kidney physiology leading to the onset of a prehypertensive phenotype in adult male offspring from gestational CPS (Mendez et al., 2019). Blood pressure regulation is a complex process, primarily governed by the kidneys. The number of nephrons decreases in multiple animal models of developmental programming, indicating that adverse conditions during nephrogenesis play a critical role in increased hypertension susceptibility in adulthood (Tain and Joles, 2015). In this context, recent evidence demonstrated that maternal administration of melatonin or agomelatine could prevent programmed hypertension in adult males gestated under continuous light. In addition, genes belonging to the renin-angiotensin system (RAS) and sodium transporters were potentially involved in programmed hypertension; interestingly, many of these effectors were differentially regulated by prenatal treatment (Tain et al., 2017b). Although, there is limited data about these effects in females. Sex differences have been observed in animal models of fetal programming, suggesting that sex hormones may be linked to a lower incidence of hypertension in females (Glen Pyle and Martino, 2018). In addition, some evidence reports sex differences in circadian sodium handling, where female rats displayed a more efficient time-of-day-dependent natriuretic response to acute salt load than males (Kittikulsuth et al., 2013). Although sex hormones have been the focus of the investigation, recent evidence demonstrated that multiple factors are involved in the complexity of sex-dependent differences in cardiovascular disease (Grigore et al., 2008; Glen Pyle and Martino, 2018).

So far, the mechanisms proposed for fetal programming of chronic disease are broad. Low-grade inflammation may provide valuable biomarkers and mechanistic insight linking gestational chronodisruption with the programming of NCDs; Melatonin exerts many functions, including modulation of inflammatory processes (Hardeland et al., 2011, 2012; Chitimus et al., 2020), in which, melatonin act as a pro and anti-inflammatory factor (Hardeland, 2019), in the present study we may speculate that melatonin modulates the inflammatory status in the offspring. Recent animal studies in developmental programming models have evidenced the potential implication of melatonin as a re-programming strategy to prevent DOHaD-related diseases (Hsu et al., 2019). Melatonin therapy has anti-oxidative, immunological, and inflammatory effects that could be prolonged until adulthood. In addition, melatonin improves placental function and directly acts on many fetal developing organs (Torres-Farfan et al., 2008; Tain and Joles, 2015; Tain et al., 2017a).

Circadian disruption chronically impairs the adequate function of different biological clocks and predisposes to pathological processes like cancer and metabolic and cardiovascular disorders (Touitou et al., 2017). Melatonin is a potent free radical scavenger, antioxidant, and cytoprotective agent playing a key protective role for the maternal–placental–fetal unit.

Besides, melatonin is involved in epigenetic regulation. For instance, the evidence demonstrated that maternal melatonin therapy up-regulates several kidney offspring genes (Tain et al., 2014) and is consistent with studies reporting inhibition of DNA methyltransferases (DNMTs) and histone deacetylases (HDACs) by melatonin (Wu et al., 2014). In the same context, we observed that in the liver from fetuses gestated under constant light, the expression of mRNAs encoding for enzymes involved in DNA methylation (Mat1a, Bhat, Bhmt2, and Ghent) was significantly decreased. Of note, prenatal melatonin treatment fully reversed this inhibitory effect of gestational chronodisruption for three of these enzymes and partially for the fourth one (Torres-Farfan et al., 2020). Hence, gestational chronodisruption modified gene expression in the three organs evaluated in female offspring, and melatonin reverted or modulated these effects. Also, we know that the intermittent rhythm of melatonin, even the absence in some periods, could link maternal photoperiod and the onset of NCD in the male offspring (Cisternas et al., 2010; Ferreira et al., 2012; Vilches et al., 2014; Salazar et al., 2018) and probably in the female offspring as well. Although we did not evaluate the effects of gestational chronodisruption on anxiety, blood pressure, and renal function, we will be assessed in future studies to establish whether these changes translate into NCD and the possible sex differences in the fetal programming by CPS; Future studies are necessary to reveal this interrogate.

## Conclusion

The pleiotropic biological functions of melatonin can generate an almost perfect temporal adaptation *in utero* for the offspring to successfully come across the day/night cycles after birth (Seron-Ferre et al., 2007; Cipolla-Neto and Do Amaral, 2018). In line with this, prenatal melatonin administration in a model of chronodisruption sustained core biorhythms in the pregnant dams and improved gestational outcomes such as gestation length and birth weight in the CPS cohort. Our findings in the adult female progeny from gestational CPS conditions are consistent with fetal programming of adrenal, cardiac, and renal physiology in the adult offspring, with multi-level consequences including molecular, endocrine, and inflammatory dysfunction and ensuing increased risk of chronic disease. The fact that prenatal melatonin treatment reversed most of these adverse effects, supports its use to protect adult offspring’s physiology from fetal programming by chronodisruption. Collectively, these results may be relevant for translation to the human clinical setting, where maternal chronodisruption can be detected and treated before or at an early stage during pregnancy to avoid deleterious effects on the progeny. Finally, further research is warranted to evaluate whether the strong protective effects of melatonin on pregnancy outcomes and offspring’s physiology are mediated by both epigenetic regulation and antioxidant effects.

## Data availability statement

The original contributions presented in this study are included in the article/supplementary material, further inquiries can be directed to the corresponding author.

## Ethics statement

The animal study was reviewed and approved by the Committee of Bioethics of the Universidad Austral de Chile (CBA# 297 and 352 UACH).

## Author contributions

NM, DH, HR, PE, MS-F, and CT-F conceived and designed the study, analyzed and interpreted the data, drafted the manuscript, critically revised important intellectual content in the manuscript, and provided overall supervision. ES-P, KV, FC, PB, and CB performed the experiments, analyzed the data, drafted the manuscript, and contributed to the intellectual content of the manuscript. All authors approved the final manuscripts and agreed to be accountable for all aspects of the work.

## Footnotes

1
www.graphpad.com
